# Methamphetamine and the hypothalamic-pituitary-adrenal axis

**DOI:** 10.3389/fnins.2015.00178

**Published:** 2015-05-27

**Authors:** Damian G. Zuloaga, Jason S. Jacobskind, Jacob Raber

**Affiliations:** ^1^Department of Psychology, University at AlbanyAlbany, NY, USA; ^2^Departments of Behavioral Neuroscience, Neurology, and Radiation Medicine, Oregon Health and Science University PortlandPortland, OR, USA; ^3^Division of Neuroscience, Oregon National Primate Research Center, Oregon Health and Science University PortlandPortland, OR, USA

**Keywords:** methamphetamine, stress, anxiety, substance abuse, glucocorticoids, HPA axis

## Abstract

Psychostimulants such as methamphetamine (MA) induce significant alterations in the function of the hypothalamic-pituitary-adrenal (HPA) axis. These changes in HPA axis function are associated with altered stress-related behaviors and might contribute to addictive processes such as relapse. In this mini-review we discuss acute and chronic effects of MA (adult and developmental exposure) on the HPA axis, including effects on HPA axis associated genes/proteins, brain regions, and behaviors such as anxiety and depression. A better understanding of the mechanisms through which MA affects the HPA axis may lead to more effective treatment strategies for MA addiction.

## Introduction

Abuse of amphetamine-like substances, including methamphetamine (MA), ranks second to cannabis in terms of worldwide illicit drug use (UNODC, 2011). A 2012 report indicates that in the United States MA is abused by approximately 439,000 people (SAMHSA, 2012), costing the United States approximately $23.4 billion annually (Nicosia et al., 2009). MA abuse is associated with high rates of relapse. A recent study indicated that 61% of MA users relapse to MA abuse within a year of release from treatment programs (Brecht and Herbeck, 2014). Furthermore, only 13% of individuals maintained abstinence from MA over the 5 year duration of the study (Brecht and Herbeck, 2014). Despite widespread need for improved MA addiction treatment options, current psychological and particularly pharmacological treatments for MA abuse have had limited success (Carson and Taylor, 2014).

Stress and the hypothalamic-pituitary-adrenal (HPA) axis are key components in psychostimulant addiction. Repeated drug use can induce functional and morphological changes in the HPA axis including alterations in stress hormone release as well as genes and proteins [e.g., corticotropin-releasing factor (CRF), arginine vasopressin (AVP), and glucocorticoid receptor (GR)] within brain regions pertinent to HPA axis function [e.g., extended amygdala, paraventricular hypothalamus (PVN); (Mantsch et al., 2007; Nawata et al., 2012)]. These changes contribute to a shift to the anti-reward pathway of drug abuse which is hypothesized to be activated secondarily to the reward pathway. This involves a change in the motivation from abusing drugs for their “pleasurable” effects to a desire to reduce internal distress (Koob et al., 2014). Emerging studies in humans indicate that the HPA axis is altered by repeated MA use, with individuals showing altered levels of stress hormones during withdrawal (Carson et al., 2012; Li et al., 2013). These alterations may contribute to adverse effects of MA, such as depression and anxiety, which in turn perpetuate further MA use. In this mini-review we summarize existing literature related to MA effects on the HPA axis and associated stress-related behaviors.

### MA activates the HPA axis

Numerous studies have demonstrated that MA activates the HPA axis (Morimasa et al., 1987; Williams et al., 2000; Zuloaga et al., 2014). Similar to stress- induced activation of the HPA axis, increased production of adrenocorticotropic hormone (ACTH) secretagogues (CRF and AVP) in neurons of the PVN is essential. When stimulated, AVP-expressing cells of the parvocellular PVN act in conjunction with CRF-expressing cells to activate cells in the anterior pituitary gland. This, in turn, stimulates cleavage of proopiomelanocortin (POMC) to produce ACTH in corticotropic cells. ACTH then enters the systemic blood stream and stimulates the adrenal cortex to release glucocorticoids (e.g., corticosterone and cortisol). Glucocorticoids circulate throughout the body and exert effects on numerous organs including the brain. This response is terminated through activation of GRs in the brain and pituitary which, via negative feedback, decrease further release of CRF and ACTH.

**Figure 1:**
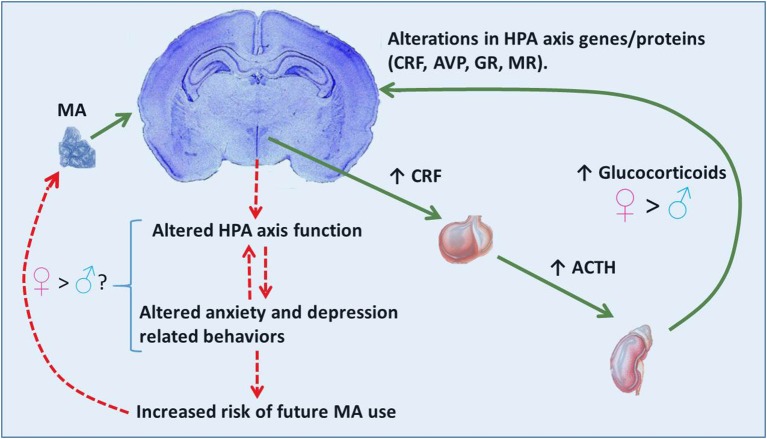
**Proposed scenario for MA effects on the HPA axis and behavior**. MA activation of the HPA axis involves release of CRF from neurosecretory cells of the paraventricular hypothalamic nucleus and subsequent release of ACTH and glucocorticoids from the anterior pituitary and adrenal cortex, respectively. Release of glucocorticoids is greater in females than males. High levels of glucocorticoids may contribute to alterations in HPA axis associated genes/proteins (CRF, AVP, GR). These effects in the brain may subsequently alter HPA axis function (release of stress hormones) and in turn affect anxiety- and depressive-related behaviors often observed during withdrawal. Altered HPA axis function and mood behaviors may provide bi-directional positive feedback, thereby exacerbating these effects. Altered mood behaviors can lead to a need to alleviate an aversive mood state, thereby increasing the risk of further MA use as a means to reduce internal distress. The relationship between the elevated glucocorticoid response to MA in females and brain and behavioral consequences are currently unknown.

Activation of the HPA is an adaptive response which prepares an organism for “fight or flight” associated situations. However, in instances of chronic activation of the HPA axis (e.g., glucocorticoid treatments, chronic stress, or abuse of certain illicit drugs) glucocorticoids can be harmful. Excessive glucocorticoid exposure can exert negative effects on the brain including: increasing cell death (Zuloaga et al., 2011), altering cell proliferation patterns (Kim et al., 2004), and inducing dendritic remodeling (Kim et al., 2014). Repeated exposure to elevated glucocorticoids can also alter brain expression of genes and proteins associated with the HPA axis including CRF, AVP, GR, and mineralocorticoid receptor (MR; Imaki et al., 1991; Reul et al., 1987; Herman et al., 1989; Sawchenko et al., 1993). These alterations contribute to lasting effects on HPA axis function. Disrupted HPA axis function, including altered glucocorticoid release, are hallmarks of depressive and anxiety spectrum disorders in humans as well as related symptomology in rodents (Carroll et al., 1968; Landgraf et al., 1999). These affective mood states have been linked to both the onset of drug use as well as relapse (Haass-Koffler et al., 2014). See Figure 1 for a hypothetical mechanism through which MA use, via hyper-activation of the HPA axis, may induce changes in HPA axis associated genes/proteins within pertinent brain regions. Disruptions in the HPA axis and related behaviors such as anxiety and depression may potentially contribute to subsequent MA use.

MA administration, ranging from 1 to 40 mg/kg, induces robust increases in corticosterone in rodents (Herring et al., 2008a; Zuloaga et al., 2014) and cortisol in nonhuman primates (Madden et al., 2005). A study in humans also reported elevated plasma cortisol in MA users that were intravenously administered a 0.5 mg/kg dose of MA (Harris et al., 2003). Recent studies in humans also indicate that chronic MA abuse can alter HPA axis function (Carson et al., 2012; Li et al., 2013). MA-dependent individuals show elevated ACTH and decreased cortisol levels at 96 h of MA abstinence compared to matched controls Li et al., 2013. These same MA-dependent individuals displayed elevated depressive symptomology (Li et al., 2013), indicating that both the HPA axis and associated behaviors are affected in recently abstinent MA abusers. Withdrawal from MA is commonly associated with elevated states of anxiety and depression (London et al., 2004; Cruickshank and Dyer, 2009), although whether these mood states are directly linked to altered HPA axis function is unknown.

In rats, chronic MA self-administration also increases anxiety and depressive-like behaviors during withdrawal from MA (Nawata et al., 2012; Jang et al., 2013). A single high dose injection of MA can also induce lasting depressive-like behavior in mice (Silva et al., 2014). Studies investigating effects of adult chronic MA on glucocorticoid release are few in number and have yielded mixed results, which may depend on timing, dose and method of administration. For example, Nawata et al. (2012) reported no alterations in blood corticosterone levels at 10 days of withdrawal from MA self-administration. However, other studies have reported lasting effects of MA on glucocorticoid levels. A neurotoxic dose of MA elevated basal corticosterone levels compared to controls at 24 h, 72 h, and 47 days after administration (Herring et al., 2008a,b; Grace et al., 2010b). Furthermore, chronic MA administration in drinking water alters the circadian rhythm of corticosterone 1 week post-withdrawal (Morimasa et al., 1987). Further studies specifically directed at investigating the HPA axis are needed to clarify the role of chronic MA on subsequent glucocorticoid release.

### Developmental MA exposure

Because the rate of MA abuse is high in women of childbearing age, there is increased interest in the developmental effects on infants prenatally exposed to MA. However, there are few investigations of either short- or long-term effects of early developmental MA exposure, particularly in the context of alterations in the HPA axis function. A 2-year follow up study of children prenatally exposed to MA found that stress-induced cortisol levels were significantly higher in MA-exposed infants (Kirlic et al., 2013). Behavioral effects that may be associated with HPA axis alterations in prenatal MA exposed human offspring include altered arousal in infants (Smith et al., 2012; Kiblawi et al., 2014) and increased emotional reactivity and anxiety/depressed behavior in 3–5 year olds (LaGasse et al., 2012; Abar et al., 2013). Polydrug abuse, domestic violence, and emotional neglect are highly associated with parental MA abuse. These confounds make it difficult to dissociate effects of MA from other variables, necessitating use of animal models to explore specific effects of MA.

Extensive brain, particularly hippocampal, development occurs during the postnatal period in the rodent, while in humans analogous development occurs prenatally (Workman et al., 2013). Due to these differences in developmental trajectory, the majority of studies involving developmental MA and the HPA axis in rodents have utilized neonatal administration. Similar to adult MA exposure, neonatal administration also induces robust elevations in blood corticosterone (Acevedo et al., 2008; Grace et al., 2008). These elevations in corticosterone are greater than those induced by severe psychological stressors such as forced swim (Grace et al., 2008). An important consideration in developmental MA models is the timing of treatment which can cause differential effects on glucocorticoid release and behavior (Williams et al., 2003; Schaefer et al., 2008). Specifically, treatment that coincides with the neonatal stress hyporesponsive period (SHRP), during which basal and stress/drug induced glucocorticoid release are naturally blunted, results in the greatest long-term deficits in learning and memory (Acevedo et al., 2007; Vorhees et al., 2009).

Although long-term deficits on learning and memory following neonatal MA exposure are well documented, lasting effects on HPA axis function and stress-related behaviors are more subtle and findings are mixed. MA exposure near the neonatal SHRP results in subtle effects on adult HPA axis responses, including a more rapid rise in plasma corticosterone after forced swim (Grace et al., 2012). However, no long term effects in plasma corticosterone were found in similarly exposed rats following adult forced confinement or challenge with MA (Grace et al., 2012). Prenatal MA exposure in rats failed to induce any effects on adult basal plasma ACTH and corticosterone levels (Cabrera et al., 1993). Furthermore, several reports indicate that neonatal MA exposure does not alter anxiety-like behaviors in adolescent or adult mice (Siegel et al., 2011; Eastwood et al., 2012). However, in rats, several neonatal MA treatment regimens result in subtle *decreases* in anxiety-like behaviors in adulthood (Williams et al., 2003; Grace et al., 2010a). On the contrary, postnatal MA exposure via breast milk induced *increased* anxiety behavior in adult rats while prenatal MA induced no behavior effects (Hruba et al., 2012). Little is known about developmental effects of MA on depression-associated behavior, although juvenile exposure increases adult depressive behavior in mice (Joca et al., 2014). Together these findings indicate that developmental effects on glucocorticoids and stress-related behaviors are subtle and highly dependent on species examined, dose, and timing of treatment.

### HPA axis associated neuropeptides and neural receptors associated with MA

Numerous studies indicate CRF regulates stress-related behaviors. Administration of CRF and genetic overexpression of CRF increase anxiety-related behaviors in rodents (Stenzel-Poore et al., 1994; Spiga et al., 2006). Conversely, administration of CRF antagonists has opposite effects, decreasing anxiety behaviors (Rassnick et al., 1993). CRF and CRF receptors (CRF-R1/CRF-R2) have also been implicated in MA abuse. CRF mRNA is elevated in the nucleus accumbens following injection with MA (Martin et al., 2012). Furthermore, exposure to a high dose of MA (10 mg/kg) enhances mRNA levels of CRF to a subsequent lesser dose of MA (2.5 mg/kg) in the rat nucleus accumbens (Cadet et al., 2014). This indicates that prior exposure to MA can induce greater elevations in CRF. Interestingly, rats that had previously self-administered MA showed elevated levels of CRF in the amygdala and plasma at 10 days of MA withdrawal (Nawata et al., 2012). These elevations in CRF were associated with increased anxiety behaviors and stress-induced reinstatement of MA-seeking (Nawata et al., 2012). The non-selective CRF receptor antagonist (α-helical CRF9–41) decreases MA-seeking behavior induced by stress (Nawata et al., 2012). Furthermore, selective CRF-R1 antagonists attenuate both cue-and MA-induced reinstatement of MA-seeking (Moffett and Goeders, 2007). These studies indicate that CRF antagonists may be effective treatments for decreasing symptoms such as anxiety, depression, and craving that are associated with MA withdrawal. Recent evidence also indicates AVP as a potential target for MA effects. Similar to CRF, prior exposure to MA can elevate subsequent MA-induced AVP mRNA in the rat nucleus accumbens (Cadet et al., 2014). Another study indicates that antagonism of an AVP receptor subtype (AVPR1b) contributes to MA-induced conditioned place preference (Subiah et al., 2012). Developmental studies indicate AVP is particularly vulnerable to MA. Exposure during neonatal and adolescent periods causes lasting reductions in AVP-expressing cells in the PVN (Zuloaga et al., 2013; Joca et al., 2014).

Other HPA axis associated proteins GR and MR are also affected by MA administration. MA exposure decreases GR, and to a lesser extent MR, in the hippocampus and hypothalamus of rodents (Lowy, 1990; Lowy and Novotney, 1994; Kabbaj et al., 2003). Within the PVN and central amygdala (CEA), there is also a robust activation of glucocorticoid receptor (GR) positive cells, identified by a significant increase in c-Fos/GR dual-labeled cells, following MA treatment. This indicates MA targets this cell phenotype in the brain and as a result may exert lasting effects on the function of these neurons. Studies involving pharmacological blockade of GR indicate that activation of GR is involved in increasing locomotor activity following MA treatment (Ago et al., 2009), although its role in other MA processes such as MA-seeking and reinstatement is unknown. See Table 1 for a summary of main findings regarding MA effects on glucocorticoids, HPA axis associated genes/proteins, and stress-related behaviors.

**Table 1:** **MA effects on glucocorticoids, HPA axis associated genes/proteins, and stress-associated behaviors**.

		**Effects**	**References**
Glucocorticoids	Adult Acute exposure	↑ levels (rodent, non-human primate, and human)	Zuloaga et al., 2014; Madden et al., 2005; Harris et al., 2003
	Adult repeated exposure	Alterations in circadian rhythm (rat)	Morimasa et al., 1987; Li et al., 2013
		Decreased basal levels during withdrawal (human)	
	Developmental acute exposure	↑ levels (mouse and rat)	Grace et al., 2008; Acevedo et al., 2008
	Developmental repeated exposure	**Neonatal exposure:** No effects or subtle ↑ following adult stressor (rat)	Grace et al., 2012
		**Prenatal Exposure:** No effect on basal levels (rat) Elevated stress induced levels in 2 year olds (human)	Cabrera et al., 1993; Kirlic et al., 2013
HPA axis associated genes/proteins	CRF	**Adult exposure:** ↑ in CEA and NAC (rat)	Nawata et al., 2012; Cadet et al., 2014
	AVP	**Neonatal/Juvenile exposure:** Lasting decreases in AVP-expressing cells in the PVN (mouse)	Zuloaga et al., 2013; Joca et al., 2014.
	GR	**Adult exposure:** ↓ in hippocampus, hypothalamus, and striatum (rat)	Lowy and Novotney, 1994
	MR	**Adult exposure:** ↓ in hippocampus (rat)	Lowy, 1990
Stress-associated behaviors	Anxiety-related	**Prenatal exposure:** No effects (rat) ↑ in 3–5 year olds (human)	Hruba et al., 2012; LaGasse et al., 2012
		**Neonatal exposure:** Subtle↑ or ↓ found in adulthood, depends on timing and dose (rat)	Williams et al., 2003; Hruba et al., 2012
		No effects (mouse)	Eastwood et al., 2012;
		**Adult exposure:** ↑ during withdrawal (rat, human)	Nawata et al., 2012; London et al., 2004
	Depressive-related	**Prenatal exposure:** ↑ in 3–5 year olds (human)	LaGasse et al., 2012
		**Juvenile exposure:** ↑ in adulthood (mouse)	Joca et al., 2014
		**Adult exposure:** ↑ during withdrawal (rat, human)	Jang et al., 2013; Li et al., 2013

Blockade of MA-induced glucocorticoid synthesis using metyrapone has been utilized to study the effects of glucocorticoids on MA-associated behaviors, particularly in the context of stress and cued reinstatement of MA use. Metyrapone administration failed to block stress induced MA seeking behavior in mice previously exposed to MA (Nawata et al., 2012). However, a combined treatment of metyrapone with the benzodiazepine oxazepam attenuated MA seeking induced by the presentation of a conditioned reinforcer (Keller et al., 2013), indicating at least some involvement of glucocorticoids in this response. In cocaine models, metyrapone reduces cocaine-induced locomotion and relapse of cocaine self-administration (Piazza et al., 1994; Marinelli et al., 1997). Little is known about corticosterone synthesis blockade on many MA-induced behaviors as it has largely been studied in the context of withdrawal.

### Neuroanatomical regions associated with MA and the HPA axis

Psychostimulant effects on the HPA axis and the anti-reward pathway of addiction are commonly associated with extended amygdala circuitry (Koob and Volkow, 2010). However, studies utilizing immediate early genes as markers of neural activation have been useful in identifying other candidate regions involved in MA regulation of the HPA axis. HPA axis associated brain areas including hypothalamic, hippocampal, cortical, and extended amygdala sub-regions show extensive immediate early gene responses following MA administration (Giardino et al., 2011; Tomita et al., 2013; Zuloaga et al., 2014). Even a relatively low dose of MA (1 mg/kg) induces a 4–5 fold increase in the number of c-Fos positive cells within the PVN and CEA, two regions central to regulation of the HPA axis. Less is known about potential alterations in neural activation patterns in HPA axis-associated brain regions following chronic MA exposure. The majority of studies involving repeated psychostimulant exposure on immediate early gene responses have focused on the neostriatum and nucleus accumbens (McCoy et al., 2011). In these regions, repeated MA blunts neural activation (McCoy et al., 2011). Another study involving MA self-administration reported that repeated MA decreases c-Fos in the CEA and BNST (Cornish et al., 2012). This finding indicates that neural activation patterns in HPA axis associated brain regions are affected by chronic MA exposure. Further understanding of the effects of repeated MA use on neural activation within these and other brain areas that regulate the HPA axis may reveal specific brain circuits associated with MA addiction.

### Sex differences in MA effects on the HPA axis

MA activation of the HPA axis is sex- dependent in neonatal and adult mice. In postnatal day 20 mice, MA induces a significant increase in corticosterone in females but not males (Acevedo et al., 2008). In adult mice, a 1 mg/kg dose of MA induces a protracted elevation in corticosterone in females compared to males (Zuloaga et al., 2014). This sex difference may be related to HPA axis negative feedback. Male mice display greater numbers of c-Fos positive and dual-labeled c-Fos/GR positive cells in two regions that regulate HPA axis negative feedback (CA3 and cingulate cortex) following the same dose of MA. Given the role of GR in regulating negative feedback (Sapolsky et al., 1985), this indicates a potential mechanism through which males confer a more rapid shutdown of the HPA axis than females. Potential sex differences in the effects of repeated MA exposure on HPA axis function are currently unknown but are likely critical in understanding sex differences in addiction associated behaviors reported in rodents and humans. These include greater MA seeking and reinstatement to MA use following a period of withdrawal in female rodents (Roth and Carroll, 2004; Reichel et al., 2012). In humans, there are numerous sex differences in MA abuse; women begin using at an earlier age than men, have a shorter duration from first use to regular use, and are more dependent on and committed to MA (Brecht et al., 2004; Dluzen and Liu, 2008; SAMHSA, 2010). Sex differences in MA-induced alterations in the HPA axis may contribute to these sexually dimorphic abuse patterns. Of particular interest is the role of gonadal steroid hormones estrogen, progesterone, and testosterone in regulating these effects. Extensive literature indicates that estrogens enhance, whereas androgens suppress, glucocorticoid release via activation of estrogen and androgen receptor subtypes (Zuloaga et al., 2008, 2011; Handa and Weiser, 2014). These same hormones regulate behavioral effects of psychostimulants such as cocaine self-administration, reinstatement, and conditioned place preference (Cooper et al., 2013; Ramoa et al., 2013; Bobzean et al., 2014). A more comprehensive understanding of the interactions between psychostimulant abuse processes, stress hormones, and gonadal steroid hormones will provide insight into the underlying causes of addiction.

### Conclusions and potential therapeutics

MA is a potent activator of the HPA axis during both development and adulthood. Prolonged release in plasma corticosterone may contribute to a variety of effects reported after MA administration. In particular, the altered expression of genes/proteins associated with the HPA axis including CRF, AVP, and GR which have been reported in a variety of HPA axis associated brain regions. These neural effects of MA have been linked to both elevated anxiety/despair states as well as increased stress-induced reinstatement to MA use. MA addiction has proven incredibly difficult to treat, particularly in terms of psychotherapeutic interventions (Carson and Taylor, 2014). Compounds that alleviate HPA axis dysfunction such as metyrapone and drugs that interact with receptors for CRF, AVP, and glucocorticoids offer alternative mechanisms of treatment. At present, only metyrapone has been assessed in the context of human MA use although pre-clinical studies on other HPA axis-associated compounds are emerging and may yield clinical trials. For these compounds, it is essential to consider sex-specific effects since both MA abuse patterns and HPA axis activation differ between the sexes.

### Conflict of interest statement

The authors declare that the research was conducted in the absence of any commercial or financial relationships that could be construed as a potential conflict of interest.
