# Erratum on: Methamphetamine and the hypothalamic-pituitary-adrenal axis

**DOI:** 10.3389/fnins.2015.00233

**Published:** 2015-06-26

**Authors:** Frontiers Production Office

**Affiliations:** Frontiers Production Office, FrontiersSwitzerland

**Keywords:** methamphetamine, stress, anxiety, substanceabuse, glucocorticoids, HPAaxis

Reason for Erratum:

Due to a misunderstanding, the last name of the author Jason S. Jacobskind was published as Jacosbskind. This error does not change the scientific conclusions of the article in any way.

The original article has been updated.

